# Assessing the Performances of Protein Function Prediction Algorithms from the Perspectives of Identification Accuracy and False Discovery Rate

**DOI:** 10.3390/ijms19010183

**Published:** 2018-01-08

**Authors:** Chun Yan Yu, Xiao Xu Li, Hong Yang, Ying Hong Li, Wei Wei Xue, Yu Zong Chen, Lin Tao, Feng Zhu

**Affiliations:** 1 Innovative Drug Research and Bioinformatics Group, School of Pharmaceutical Sciences and Collaborative Innovation Center for Brain Science, Chongqing University, Chongqing 401331, China; yucy@cqu.edu.cn (C.Y.Y.); lixiaoxu@cqu.edu.cn (X.X.L.); yangh0921@cqu.edu.cn (H.Y.); liyh@cqu.edu.cn (Y.H.L.); xueww@cqu.edu.cn (W.W.X.); 2 Innovative Drug Research and Bioinformatics Group, College of Pharmaceutical Sciences, Zhejiang University, Hangzhou 310058, China; 3 Bioinformatics and Drug Design Group, Department of Pharmacy, and Center for Computational Science and Engineering, National University of Singapore, Singapore 117543, Singapore; 20121802134@cqu.edu.cn; 4 School of Medicine, Hangzhou Normal University, Hangzhou 310012, China; linntaoo@hotmail.com

**Keywords:** false discovery rate, machine learning, protein function prediction, support vector machine, BLAST

## Abstract

The function of a protein is of great interest in the cutting-edge research of biological mechanisms, disease development and drug/target discovery. Besides experimental explorations, a variety of computational methods have been designed to predict protein function. Among these in silico methods, the prediction of BLAST is based on protein sequence similarity, while that of machine learning is also based on the sequence, but without the consideration of their similarity. This unique characteristic of machine learning makes it a good complement to BLAST and many other approaches in predicting the function of remotely relevant proteins and the homologous proteins of distinct function. However, the identification accuracies of these in silico methods and their false discovery rate have not yet been assessed so far, which greatly limits the usage of these algorithms. Herein, a comprehensive comparison of the performances among four popular prediction algorithms (BLAST, SVM, PNN and KNN) was conducted. In particular, the performance of these methods was systematically assessed by four standard statistical indexes based on the independent test datasets of 93 functional protein families defined by UniProtKB keywords. Moreover, the false discovery rates of these algorithms were evaluated by scanning the genomes of four representative model organisms (*Homo sapiens*, *Arabidopsis thaliana*, *Saccharomyces cerevisiae* and *Mycobacterium tuberculosis*). As a result, the substantially higher sensitivity of SVM and BLAST was observed compared with that of PNN and KNN. However, the machine learning algorithms (PNN, KNN and SVM) were found capable of substantially reducing the false discovery rate (SVM < PNN < KNN). In sum, this study comprehensively assessed the performance of four popular algorithms applied to protein function prediction, which could facilitate the selection of the most appropriate method in the related biomedical research.

## 1. Introduction

The function of a protein is of great interest in the current research of biological mechanisms [1], disease development [2] and drug/target discovery [3,4,5,6,7], and a variety of databases is available for providing functional annotations from the perspectives of the sequence [8], protein-protein interaction [9,10], the biological network [11,12,13,14,15] and many specific functional classes [16,17,18,19,20,21,22]. However, a substantial gap is still observed between the total number of protein sequences discovered and that of proteins characterized with known function [23]. To cope with this gap, thousands of high-throughput genome projects are under study [24], and over 13 million sequences have been discovered, but only 1% of these validated by experimental annotation [25]. Apart from those experimental approaches, many in silico methods have been designed and extensively used to discover protein functions [26]. These include clustering of sequences [27], gene fusion [28], sequence similarity [29,30], evolution study [31], structural comparison [32], protein-protein interaction [33,34], functional classification via the sequence-derived [35,36,37,38] and domain [39,40,41,42,43] feature, omics profiling [44,45,46,47] and integrated methods, which collectively consider multiple methods and data to promote the performance of function prediction [48,49,50,51].

Among these in silico methods [52], the basic local alignment search tool (BLAST) [53] revealing protein functions based on excess sequence similarity [54] demonstrated great capacity and attracted substantial interest from the researchers of this field [55,56]. Apart from BLAST, machine learning algorithms have been frequently applied in recent years for functional prediction [57,58,59,60,61,62], and a variety of online software tools based on machine learning was developed as predictors without considering the similarity in sequence or structure [36,63]. This unique characteristic makes machine learning a good complement to other in silico approaches in predicting the function of remotely relevant protein and the homologous proteins of distinct functions [64,65].

So far, three machine learning algorithms, including K-nearest neighbor (KNN), probabilistic neural network (PNN) and support vector machine (SVM), have been extensively explored to classify proteins into certain functional families by analyzing the sequence-based physicochemical property [64,65] and to assess protein functional classes collectively [63]. These algorithms are recognized as powerful alternative methods for predicting the function of both proteins [66,67,68,69,70] and other molecules [71]. However, over one third of the protein sequences in UniProt [26] are still labeled as “putative”, “uncharacterized”, “unknown function” or “hypothetical”, and the difficulty in discovering the function of the remaining proteins is reported to come mainly from the false discovery rate of in silico algorithms [55,56,72]. Moreover, the identification accuracies of those approaches still need to be further improved [55,56,73]. Thus, it is urgently needed to assess the identification accuracies and false discovery rates among those different in silico approaches.

In this study, the performances of four popular functional prediction algorithms (BLAST, SVM, KNN and PNN) were comprehensively evaluated from two perspectives. In particular, the identification accuracies (measured by four standard statistical indexes) of various algorithms were systematically evaluated based on the independent test data of 93 functional families. Secondly, the false discovery rates of these algorithms were compared by scanning the genomes of four representative model species (*Homo sapiens*, *Arabidopsis thaliana*, *Saccharomyces cerevisiae* and *Mycobacterium tuberculosis*). In sum, these findings provided detailed information on the performances of those algorithms that are popular for protein function prediction, which may facilitate the choice of the appropriate algorithm(s) in the related biomedical research.

## 2. Results and Discussion

### 2.1. Assessment of the Identification Accuracies Measured by Four Popular Metrics

**Figure 1:**
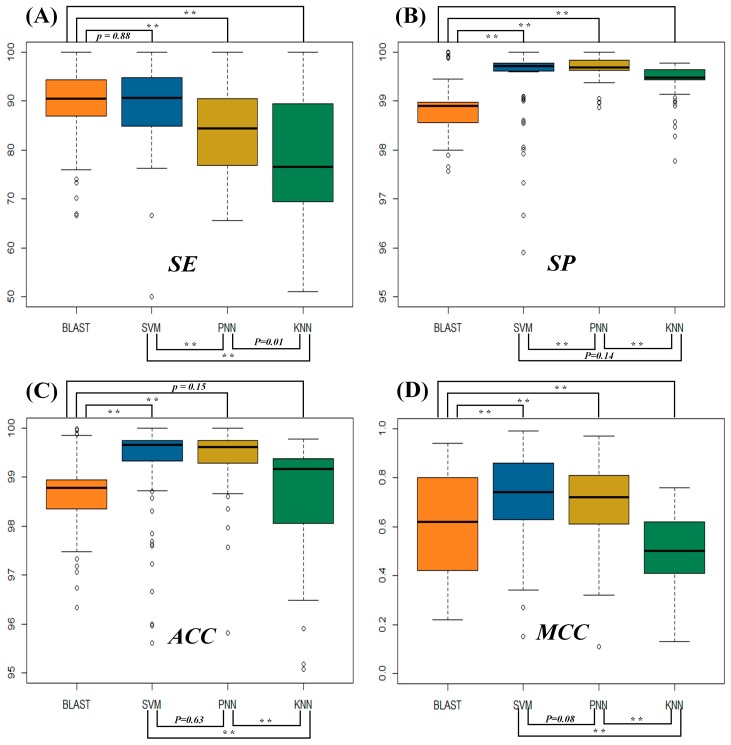
Statistical differences in the performance of four protein function prediction algorithms (BLAST, SVM, PNN and KNN) assessed by four metrics: (**A**) sensitivity (*SE*); (**B**) specificity (*SP*); (**C**) accuracy (*ACC*); and (**D**) Matthews correlation coefficient (*MCC*). Significant and moderately significant differences were shown by a *p*-value of <0.01 (**), respectively.

The statistical differences in sensitivity (*SE*) (Figure 1A), specificity (*SP*) (Figure 1B), accuracy (*ACC*) (Figure 1C) and Matthews correlation coefficient (*MCC*) (Figure 1D) among four popular functional prediction algorithms are illustrated. As illustrated in Figure 1A, the *SE* of BLAST measured by the independent test dataset of 93 families was roughly equivalent to that of SVM, but statistically higher than that of both PNN and KNN. In particular, the *SE* of 93 functional families was 50.00~99.99% for SVM, 43.93~99.99% for BLAST, 65.52~99.99% for PNN and 51.09~99.99% for KNN, and the *SE* median values of BLAST, SVM, PNN and KNN equaled 90.59%, 90.52%, 84.38% and 76.54%, respectively. As shown in Figure 1B, the majority of the *SPs* of all algorithms surpassed 98.00%; *SPs* of 93 functional families were 95.90~99.99% for SVM, 97.56~99.99% for BLAST, 98.87~99.99% for PNN and 97.77~99.43% for KNN; and the *SP* median value of BLAST, SVM, PNN and KNN was 98.90%, 99.72%, 99.67% and 99.44%, respectively. These results revealed a relatively low level of false discovery rates for all popular functional prediction algorithms.

Due to the dominant number of negative samples in the independent test datasets, the statistical difference in *ACC* was very similar to that of *SP* (Figure 1C). The majority of the *ACCs* of all algorithms surpassed 97%. The *ACCs* of 93 functional families were between 95.61% and 99.99% for SVM, between 66.68% and 99.98% for BLAST, between 95.81% and 99.99% for PNN and between 81.39% and 99.77% for KNN. Moreover, median values of *ACCs* of BLAST, SVM, PNN and KNN equaled 98.78%, 99.66%, 99.61% and 99.16%, respectively. *MCC* was frequently applied to reflect the stability of the protein function predictor and was considered as one of the most comprehensive parameters because of its full consideration of TP, TN, FP and FN. As shown in Figure 1D, the *MCC* of both SVM and PNN was better than that of BLAST and KNN. The majority of *MCCs* were over 0.6 and 0.4 for SVM-PNN and BLAST-KNN, respectively. In particular, *MCCs* of 93 functional families were between 0.15 and 0.99 for SVM, between 0.22 and 0.94 for BLAST, between 0.11 and 0.97 for PNN and between 0.13 and 0.76 for KNN. The median values of *MCCs* for BLAST, SVM, PNN and KNN equaled 0.62, 0.74, 0.72 and 0.50, respectively. In sum, there were consistently low levels of the false discovery rate among all algorithms as assessed by the metric *SP*. However, when the positive discovery rates (*SEs*) and the stability of prediction (*MCC*) were considered, SVM, PNN and BLAST stood out as more powerful algorithms for protein function prediction.

### 2.2. Evaluating the Statistical Differences in SE and MCC among Four Metrics

For the machine learning algorithms (SVM, PNN and KNN), there was a significant statistical difference in their *SEs* and *MCCs*. As shown in Figure 1A, the statistical difference in SEs between SVM and PNN equaled 3.5 × 10^−6^, while that between SVM and KNN was 1.0 × 10^−11^. Moreover, there was a significant statistical difference between PNN and KNN (*p*-value = 0.01). In particular, the number of families with *SEs* of >90%, ≤90% and >80% and ≤80% for SVM equaled 49, 33 and 11, respectively; the number of families with *SEs* of >90%, ≤90% and >80% and ≤80% for PNN equaled 17, 25 and 20, respectively; and the number of functional families with *SEs* of >90%, ≤90% and >80% and ≤80% for KNN equaled 19, 13 and 45, respectively. Similar to the *SE*, the statistical difference in *MCC* between SVM and PNN was 0.08, and that between SVM and KNN was 2.2 × 10^−16^. Moreover, there was a clear statistical difference between PNN and KNN (*p*-value = 2.2 × 10^−16^). In particular, the number of families with *MCCs* of >0.85, ≤0.85 and >0.7 and ≤0.7 for SVM was 26, 26 and 41, respectively; the number of functional families with *MCCs* of >0.85, ≤0.85 and >0.7 and ≤0.7 for PNN equaled 6, 29 and 27, respectively; and there were no protein families with *MCCs* over 0.7 for KNN. In summary, there were clear ascending trends in both *SE* and *MCC* as shown in Figure 1A,D (from KNN to PNN to SVM).

Similar to SVM, BLAST also demonstrated great performances in both *SE* and *MCC*. The statistical differences (measured by *p*-value) in the *SE* and *MCC* between BLAST and SVM were 0.88 and 2.0 × 10^−7^, respectively. As demonstrated in Table 1 and Table S1, the *SE* of BLAST surpassed that of SVM in 51 families, but was worse than that of SVM in 40 families. Moreover, the *SEs*’ median values (90.52% for BLAST and 90.59% for SVM) and mean values (88.92% for BLAST and 89.08% for SVM) indicated that the *SE* of SVM was slightly better than that of BLAST and significantly better than that of PNN and KNN. Meanwhile, *MCC* of SVM was higher than that of BLAST in 68 families, but was lower than that of BLAST in 20 families. The *MCCs*’ median values (0.62 for BLAST, 0.74 for SVM) and mean values (0.61 for BLAST, 0.73 for SVM) indicated a slight improvement in prediction stabilities by SVM.

The amphibian defense peptide family (KW-0878; KW, keyword) was the family with the highest *SE* (99.99%) for SVM, BLAST and KNN, which was known to be a rich source of antimicrobial peptides with a broad spectrum of antimicrobial activities against pathogenic microorganisms [74,75,76]. The superior *SE* of this family may come from its nature as a conserved element of the defense system of various species [77].

### 2.3. In-Depth Assessment of the False Discovery Rate by Genome Scanning

**Figure 2:**
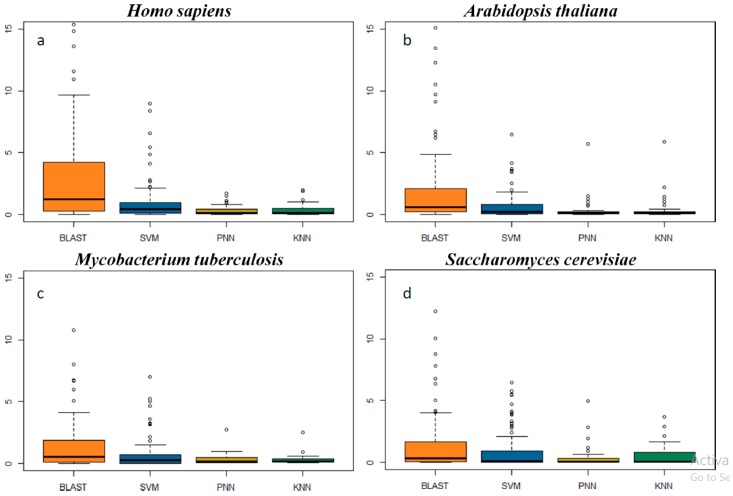
The false discovery rates reflected by the percentage of proteins identified from the genomes of (**a**) *Homo sapiens*, (**b**) *Arabidopsis thaliana*, (**c**) *Saccharomyces cerevisiae* and (**d**) *Mycobacterium tuberculosis*.

Genome scanning has been frequently used to evaluate the false discovery rate of function prediction tools [78,79]. To have a comprehensive understanding of methods’ false discovery rate, the genomes of four model organisms representing four kingdoms (*Homo sapiens* from Animalia, *Arabidopsis thaliana* from Plantae, saccharomyces cerevisiae from Fungi and *Mycobacterium tuberculosis* from Bacteria) were collected. As demonstrated in Table 2 and Table S2, the genome scanning revealed that the number of proteins in any of those 93 studied families predicted by SVM, PNN and KNN did not exceed 10% of the total number of proteins in the whole genome, and this was the same situation for the majority (82%) of the 93 studied families by BLAST. The higher number of proteins predicted for a certain functional family may indicate a higher false discovery rate [78,79]. For the human genome, the number of proteins identified by SVM was equivalent to or was slightly higher than that of both PNN and KNN, but was significantly lower than that of BLAST (Figure 2a). In addition, the proteins identified by PNN were lower than that of KNN in 11 families and higher in 20 families.

**Figure 3:**
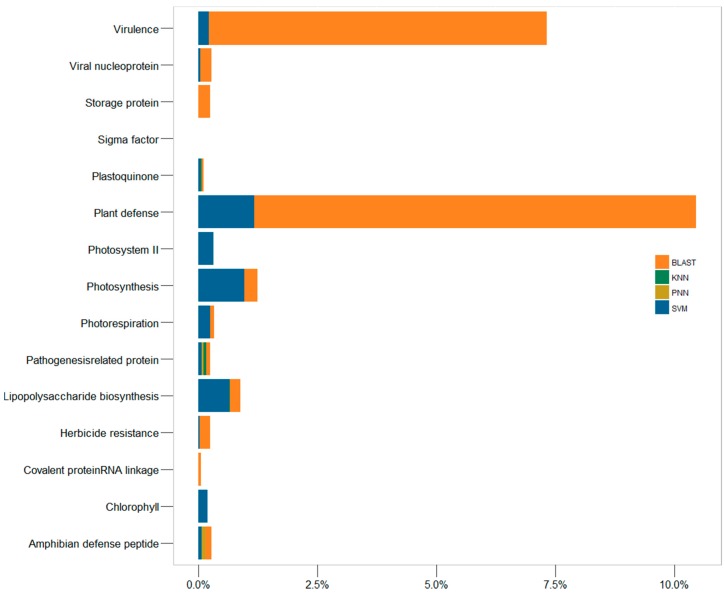
The false discovery rates reflected by the percentage of proteins of 15 protein families only existing in plants, microbes or viruses, but not existing in the human genome identified from the genomes of *Homo sapiens*.

Moreover, 15 protein families only existing in plants, microbes or viruses (Table S3, not existing in the human genome) were collected for assessing the false discovery rate of each algorithm. For example, the covalent protein-RNA linkage family (KW-0191) contained proteins attaching covalently to the RNA molecules in virus [80], and the storage protein (KW-0758) included the proteins as a source of nutrients for the development or growth of the organism in plants. For these families (Table S3), SVM did not identify any proteins from the human genome, while 0.06% and 0.25% of the proteins in the human genome were falsely assigned by BLAST to the family of covalent protein-RNA linkage protein and storage protein, respectively. As illustrated in Figure 3, several other families (such as plant defense, virulence) also demonstrated a significantly higher false discovery rate by BLAST than that of SVM.

For the other three genomes, their situation was similar to the human genome. Take the *Arabidopsis thaliana* genome as an example: proteins identified by SVM were equivalent to or slightly higher than those by PNN and KNN in all protein families, but lower than that of BLAST in 77 families, and the number of protein discovered by PNN was lower than that of KNN in 26 families. In summary, the level of false discovery rate (Figure 2b–d) could be ordered as BLAST > SVM > PNN and KNN. These results revealed that BLAST was more prone to generate a false discovery rate than the other three machine learning methods (SVM > PNN ≈ KNN).

As reported [81,82,83,84,85], an open web-server is recognized as useful for constructing effective methods and tools. A variety of web-servers have increasing impacts on medical sciences [86], driving medicinal chemistry to an unprecedented revolution [87], and efforts will be further made to develop web-based services for the performance assessment discussed in this study.

## 3. Materials and Methods

To construct a valid statistical model for a biology problem based on protein sequences [88,89,90,91,92,93,94,95,96,97], a rule of five steps is needed [98]. Firstly, a valid construction of datasets for both training and testing the model is required. Secondly, an effective conversion of the sequence to the digital feature vector is asked to represent their targeted properties. Thirdly, a powerful statistical method should be designed for the functional prediction. Fourthly, the accuracies of the constructed statistic model should be validated correctly. Fifthly, a web-server based on the constructed model may be further developed for public access. The corresponding methods and steps adopted in this study are provided and described below.

### 3.1. Collecting the Protein Sequences of Different Functional Families

Table 1 provides a full list of 93 protein families collected from UniProt [43], and the performances of the popular protein function prediction methods (BLAST, KNN, PNN, SVM) were measured via independent test datasets (the way to generate an independent dataset is shown in the following Section 2.2). These 93 included 12 families of binding molecules (e.g., sodium-, potassium-, SH3- and RNA-binding), 15 ligand families (e.g., plastoquinone ligand, vitamin C ligand and ubiquinone ligand), 58 families defined by Gene Ontology (40 molecular functions and 18 biological processes) and 8 broad families defined by UniProt [43]. All families were contained in the keyword categories of UniProt, and the majority (82.7%) of these 93 families were able to be mapped to GO terms (Table 1). Protein entries that have not been manually annotated and reviewed by UniProtKB curators in a keyword category were not considered for analysis in this study. As a result, 107~49,517 protein-entries from 93 families were collected.

### 3.2. Construction of the Training and Testing Datasets

The independent test dataset was frequently constructed to evaluate the performances of protein function predictors in recent years [99,100,101,102,103,104]. To construct a valid set of data for building the predictor of each family, the datasets of the training, testing and independent test were generated by a strictly defined process after the data collection described in Section 2.1. Firstly, all proteins of different sequences in a specific family are assigned randomly with a number, which is within the range of the total number of proteins in that family. Secondly, these sequences in each protein function family were sequentially selected based on the number assigned and then iteratively added to the training, testing and independent test datasets. Samples in these datasets are all known as the positive samples. Thirdly, the Pfam families [16] of the proteins of a certain functional family were retrieved from the Pfam database [16] for generating negative samples. The Pfam family with protein(s) of this functional family was defined as the “positive” one, and the remaining families were grouped into the “negative” ones. Finally, 3 representatives were randomly picked out of the negative families and sequentially added to the training, testing and independent test datasets, and samples in these datasets are thus known as the negative samples. It is necessary to emphasize that there was no overlap among the datasets of the training, testing and independent test [60,61].

To assess the false discovery rate among algorithms, the genomes of four model organisms representing four kingdoms (*Homo sapiens* from Animalia, *Arabidopsis thaliana* from Plantae, *Saccharomyces cerevisiae* from Fungi and *Mycobacterium tuberculosis* from Bacteria) were collected from UniProt. The protein entries without any manual annotation and review by the UniProtKB curators were not taken into consideration. In total, 20,183, 15,169, 6721 and 2166 protein sequences in FASTA format were collected for human, *Arabidopsis thaliana*, *Saccharomyces cerevisiae* and *Mycobacterium tuberculosis*, respectively.

### 3.3. Feature Vectors Used for Representing the Protein Sequence

The conversion of the protein sequence into the digital feature vector was conducted based on properties of each residue within that protein. These properties include: (1) charge; (2) polarizability; (3) polarity; (4) surface tension; (5) amino acid (AA) composition; (6) van der Waals volume via normalizing; (7) hydrophobicity; (8) solvent accessibility; and (9) protein secondary structure [36,105,106,107]. Then, 3 features were applied to describe each property [36]. These features contained: (a) composition (No. of AAs of a particular property over the total No. of AAs; (b) transition (the percentage of AAs with a certain property was followed by AAs with a different property); and (c) distribution (the sequence lengths within which the first, one fourth, half, three-quarters and all of the AAs of specific property were localized). The detailed procedure for generating the feature vector from the sequence was described in previous publications [36,65]. These features have already been successfully applied to facilitate the prediction of enzyme functional [108] and structural classes [107].

### 3.4. Functional Prediction of Protein Constructed by Machine Learning

To construct the prediction model, the parameters of machine learning methods were optimized using the testing dataset for each training process. Once suitable parameters were discovered, a new training set was constructed by combining the original training and testing datasets, and the corresponding parameters were directly accepted for training a new model. To assess the performance of the constructed models and detect possible over-fitting, the independent test set was further applied. It is necessary to emphasize that all duplicates in the protein sequence were removed during datasets’ construction.

### 3.5. Construction of Protein Functional Prediction Model Based on Sequence Similarity

Sequence similarity was assessed by the NCBI Protein-Protein BLAST (Version 2.6.0+) [53,54]. Firstly, the combined training and testing dataset was adopted to form the BLAST database, and the sequences in the independent test dataset were used as queries. The BLAST E-value and percentage sequence identity were usually applied to represent the level of similarity between sequences [109]. The functional variation between proteins was reported to be rare when their sequence identity was more than 40% [110,111]. Thus, an E-value of 0.001 and a sequence identity of 40% were adopted as the cutoffs in this study to assess the functional conservation of BLAST hits.

### 3.6. Assessing the Identification Accuracies of the Studied Methods

The performance of protein function prediction algorithms was systematically assessed by four popular metrics, sensitivity (*SE*), specificity (*SP*), accuracy (*ACC*) and Matthews correlation coefficient (*MCC*), based on the independent test datasets generated from the 93 studied families (Supplementary Materials Table S1). All 4 metrics were widely used in assessing the performance of protein function predictors [112,113,114,115,116,117]. In particular, *SE* is defined by the percentage of true positive samples correctly identified as “positive” [118,119] (shown in Equation (1)):

(1) $$SE=\frac{\mathrm{TP}}{\mathrm{TP}+\mathrm{FN}}$$

*SP* indicates the proportion of true negative samples that were correctly predicted as “negative” [118,119] (in Equation (2)):

(2) $$SP=\frac{\mathrm{TN}}{\mathrm{TN}+\mathrm{FP}}$$

*ACC* refers to the number of true samples (positive plus negative) divided by the number of all samples studied (shown in Equation (3)):

(3) $$ACC=\frac{\mathrm{TP}+\mathrm{TN}}{\mathrm{TP}+\mathrm{FN}+\mathrm{TN}+\mathrm{FP}}$$

The *MCC* was an important metric reflecting the stability of a protein function predictor, which described the correlation between a predictive value and an actual value [118,119]. It has been considered as one of the most comprehensive parameters in any category of predictors due to its full consideration of all four results. In particular, the *MCC* could be calculated by Equation 4:

(4) $$MCC=\frac{\left(\mathrm{TP}*\mathrm{TN}-\mathrm{FP}*\mathrm{FN}\right)}{\sqrt{\left(\mathrm{TP}+\mathrm{FN}\right)*\left(\mathrm{TP}+\mathrm{FP}\right)*\left(\mathrm{TN}+\mathrm{FP}\right)*\left(\mathrm{TN}+\mathrm{FN}\right)}}$$

In particular, those four results were TP (No. of true positive samples), TN (No. of true negative samples), FP (No. of false positive samples) and FN (No. of false negative samples) [118,119]. It is very important to emphasize that these four metrics are applicable to the single-class situations (each protein is grouped into just one family). For the multi-class situations frequently observed in complicated biological networks [81,82,83,84] and biomedical researches [84,89,117], different metrics should be defined [120].

### 3.7. The Rates of False Discovery of the In Silico Methods Studied Here

As reported, genome scanning was a comprehensive method to evaluate the capacity of protein functional prediction tools in identifying and classifying protein families [78,79]. In this paper, an evaluation of the false discovery rate of the studied protein function predictors was performed by scanning the genomes of 4 model organisms representing 4 kingdoms (*Homo sapiens* from Animalia, *Arabidopsis thaliana* from Plantae, *Saccharomyces cerevisiae* from Fungi and *Mycobacterium tuberculosis* from Bacteria). The false discovery rates were assessed by reconstructing the prediction models of those in silico algorithms. In particular, the sequences of proteins in a certain functional family were all put into the reference database for BLAST scanning and were also used to reconstruct the machine learning models using the optimized parameters obtained in Section 3.4. In reality, the total amount of proteins not belonging to a certain family should be much larger than that of proteins in that family. Therefore, a tiny reduction in the value of *SP* may lead to a significant discovery of false positive hits, which reminded us to use *SP* as an effective indicator when evaluating the model’s false discovery rates.

## 4. Conclusions

This study discovered substantially higher sensitivity (*SP*) and stability (*MCC*) of BLAST and SVM than that of PNN and KNN. However, the machine learning algorithms (PNN, KNN and SVM) were found capable of significantly reducing the false discovery rate (with PNN and KNN performed the best). In conclusion, this study comprehensively assessed the performances of popular algorithms applied to protein function prediction, which could facilitate the selection of the appropriate method in the related biomedical research.

## Figures and Tables

**Table 1:** The performance of four protein function prediction algorithms assessed by four popular metrics: sensitivity (*SE*), specificity (*SP*), accuracy (*ACC*) and Matthews correlation coefficient (*MCC*).

UniProt Keyword	Protein Functional Family	GO Category	BLAST				SVM				PNN				KNN			
			*SE %*	*SP %*	*AC %*	*MCC*	*SE %*	*SP %*	*AC %*	*MCC*	*SE %*	*SP %*	*AC %*	*MCC*	*SE %*	*SP %*	*AC %*	*MCC*
KW-0020	Allergen	-	76.32	98.92	98.78	0.48	84.81	99.69	99.66	0.57	86.42	99.84	99.81	0.69	74.07	99.48	99.32	0.41
KW-0049	Antioxidant	GO:0016209	94.15	99.23	99.20	0.60	89.00	99.76	99.73	0.67	86.00	99.84	99.80	0.71	69.00	99.42	99.24	0.43
KW-0117	Actin capping	GO:0051693	94.55	99.08	99.07	0.35	93.98	99.75	99.74	0.70	91.18	99.80	99.78	0.71	73.53	99.42	99.22	0.43
KW-0147	Chitin-binding	GO:0008061	86.96	98.96	98.94	0.34	91.75	99.72	99.68	0.78	75.36	99.61	99.47	0.63	93.84	98.57	98.05	0.37
KW-0157	Chromophore	GO:0018298	96.70	98.54	98.51	0.70	93.83	99.74	99.68	0.86	86.91	99.66	99.52	0.80	89.38	99.48	98.53	0.59
KW-0195	Cyclin	GO:0061575	89.34	98.92	98.89	0.44	97.96	99.78	99.78	0.60	89.80	99.84	99.83	0.62	75.51	99.63	99.53	0.39
KW-0251	Elongation factor	GO:0003746	99.51	98.57	98.60	0.83	96.72	99.67	99.62	0.92	84.14	99.67	99.29	0.85	95.84	99.46	97.21	0.63
KW-0339	Growth factor	GO:0008083	94.05	98.99	98.95	0.65	84.30	99.69	99.62	0.76	86.01	99.81	99.72	0.80	76.54	99.66	99.16	0.61
KW-0343	GTPase activation	GO:0005096	76.06	98.57	98.40	0.47	92.45	99.67	99.65	0.66	86.73	99.82	99.78	0.72	61.95	99.44	99.25	0.46
KW-0344	Guanine-nucleotide releasing factor	GO:0005085	74.09	98.57	98.44	0.39	83.33	99.72	99.69	0.57	89.74	99.64	99.62	0.56	93.59	99.15	98.95	0.31
KW-0396	Initiation factor	GO:0003743	96.88	98.92	98.86	0.83	91.36	99.66	99.50	0.87	74.21	99.82	99.32	0.81	77.64	99.45	97.98	0.65
KW-0497	Mitogen	GO:0051781	89.25	98.98	98.96	0.40	92.74	99.73	99.66	0.85	83.60	99.61	99.45	0.75	85.22	99.62	98.78	0.62
KW-0505	Motor protein	GO:0098840	93.38	98.96	98.91	0.63	89.47	99.75	99.72	0.69	80.70	99.86	99.80	0.72	64.04	99.45	99.25	0.46
KW-0514	Muscle protein	-	94.22	98.95	98.92	0.57	95.38	99.75	99.73	0.74	89.23	99.69	99.65	0.67	80.00	99.60	99.32	0.51
KW-0515	Mutator protein	GO:1990633	97.65	98.97	98.97	0.42	83.82	99.79	99.76	0.60	77.94	99.84	99.80	0.61	70.59	99.45	99.32	0.38
KW-0568	Pathogenesis related protein	GO:0009607	92.86	98.98	98.97	0.29	93.36	99.78	99.74	0.89	94.87	99.63	99.58	0.84	91.20	99.71	98.72	0.64
KW-0734	Signal transduction inhibitor	GO:0009968	81.25	98.97	98.94	0.31	84.62	99.71	99.69	0.45	84.62	99.68	99.66	0.43	87.18	99.63	99.54	0.34
KW-0786	Thiamine pyrophosphate binding	-	97.08	98.95	98.93	0.71	96.04	99.73	99.70	0.85	87.70	99.89	99.79	0.87	74.76	99.43	98.80	0.58
KW-0830	Ubiquinone binding	-	98.37	98.50	98.49	0.87	94.07	99.72	99.56	0.92	82.58	99.46	98.98	0.82	91.47	99.73	97.20	0.68
KW-0847	Vitamin C binding	GO:0031418	94.21	98.96	98.94	0.46	91.89	99.79	99.78	0.53	97.30	99.69	99.69	0.48	81.08	99.64	99.56	0.35

**Table 2:** The false discovery rate assessed by the percentage of proteins identified from human and *thaliana* genomes by different algorithms.

UniProt Keyword	Protein Functional Family	Homo Sapiens					Arabidopsis Thaliana				
		UniProt (%)	SVM (%)	BLAST (%)	PNN (%)	KNN (%)	UniProt (%)	SVM (%)	BLAST (%)	PNN (%)	KNN (%)
KW-0117	Actin capping	0.09	0.12	0.72	0.10	0.10	0.05	0.07	0.11	0.05	0.05
KW-0020	Allergen	0.02	0.18	3.68	0.11	0.04	0.01	0.17	6.22	0.07	0.09
KW-0049	Antioxidant	0.07	0.09	0.50	0.08	0.07	0.09	0.16	1.11	0.12	0.13
KW-0147	Chitin-binding	0.02	0.16	0.36	0.02	0.10	0.08	0.24	3.57	0.08	0.18
KW-0157	Chromophore	0.07	0.15	2.10	0.07	0.10	0.28	0.38	0.88	0.23	0.30
KW-0195	Cyclin	0.16	0.24	0.40	0.18	0.19	0.33	0.36	0.61	0.34	0.34
KW-0251	Elongation factor	0.08	0.11	0.45	0.08	0.09	0.15	0.19	0.48	0.14	0.16
KW-0339	Growth factor	0.65	0.93	2.50	0.71	0.73	0.12	0.18	0.24	0.13	0.14
KW-0343	GTPase activation	0.97	1.19	5.47	0.93	1.02	0.28	0.24	1.36	0.21	0.23
KW-0344	Guanine-nucleotide releasing factor	0.73	0.86	5.37	0.73	0.75	0.18	0.20	2.12	0.17	0.19
KW-0396	Initiation factor	0.24	0.39	1.70	0.26	0.25	0.26	0.38	1.71	0.24	0.28
KW-0497	Mitogen	0.20	0.65	4.37	0.30	0.35	0.00	0.07	0.52	0.01	0.02
KW-0505	Motor protein	0.66	0.75	4.07	0.67	0.67	0.59	0.45	2.14	0.34	0.42
KW-0514	Muscle protein	0.31	0.42	4.35	0.37	0.39	0.00	0.17	1.26	0.11	0.13
KW-0515	Mutator protein	0.01	0.02	0.05	0.01	0.01	0.01	0.01	0.05	0.01	0.01
KW-0568	Pathogenesis-related protein	0.00	0.08	0.09	0.04	0.05	0.13	0.20	0.91	0.15	0.16
KW-0734	Signal transduction inhibitor	0.22	0.23	1.22	0.21	0.21	0.01	0.01	0.74	0.01	0.01
KW-0786	Thiamine pyrophosphate binding	0.06	0.07	0.13	0.06	0.06	0.12	0.15	0.28	0.13	0.14
KW-0830	Ubiquinone binding	0.08	0.71	0.12	0.19	0.60	0.13	0.25	0.42	0.17	0.18
KW-0847	Vitamin C binding	0.10	0.12	0.18	0.10	0.09	0.07	0.11	0.53	0.07	0.08

## Supplementary Materials

The Supplementary Materials are available online at www.mdpi.com/1422-0067/19/1/183/s1.
