# Supplementary material for: Assessing the Performances of Protein Function Prediction Algorithms from the Perspectives of Identification Accuracy and False Discovery Rate
Source: Int J Mol Sci. 2018 Jan 8;19(1):183. doi: 10.3390/ijms19010183 (PMC5796132; doi:10.3390/ijms19010183)
Supplement: Supplementary file 1 [file ijms-19-00183-s001.zip › Zhu-Supplementary Table S3.docx]

**Table S3**. The percentage of predicted proteins in human genome across the 15 non-human protein families

| **Uniprot keyword** | **Protein family** | **Uniprot** (%) | **SVM** (%) | **BLAST**  (%) | **PNN** (%) | **KNN** (%) |
| --- | --- | --- | --- | --- | --- | --- |
| KW-0731 | Sigma factor | 0 | 0 | 0 | - | - |
| KW-0191 | Covalent protein-RNA linkage | 0 | 0 | 0.06 | - | - |
| KW-0758 | Storage protein | 0 | 0 | 0.25 | - | - |
| KW-0359 | Herbicide resistance | 0 | 0.04 | 0.21 | - | - |
| KW-0543 | Viral nucleoprotein | 0 | 0.05 | 0.23 | - | - |
| KW-0618 | Plastoquinone | 0 | 0.05 | 0.04 | - | 0.03 |
| KW-0568 | Pathogenesis-related protein | 0 | 0.08 | 0.09 | 0.04 | 0.05 |
| KW-0878 | Amphibian defense peptide | 0 | 0.08 | 0.12 | 0.08 | - |
| KW-0148 | Chlorophyll | 0 | 0.2 | 0 | - | - |
| KW-0843 | Virulence | 0 | 0.23 | 7.09 | - | - |
| KW-0601 | Photorespiration | 0 | 0.25 | 0.09 | - | - |
| KW-0604 | Photosystem II | 0 | 0.33 | 0 | - | - |
| KW-0448 | Lipopolysaccharide biosynthesis | 0 | 0.65 | 0.22 | - | 0.02 |
| KW-0602 | Photosynthesis | 0 | 0.98 | 0.27 | - | - |
| KW-0611 | Plant defense | 0 | 1.18 | 9.28 | - | - |
